# NADPH Oxidase Inhibitor Apocynin Attenuates PCB153-Induced Thyroid Injury in Rats

**DOI:** 10.1155/2016/8354745

**Published:** 2016-03-07

**Authors:** Ablikim Abliz, Chen Chen, Wenhong Deng, Weixing Wang, Rongze Sun

**Affiliations:** Department of General Surgery, Renmin Hospital of Wuhan University, 238 Jiefang Road, Wuhan, Hubei 430060, China

## Abstract

PCBs, widespread endocrine disruptors, cause the disturbance of thyroid hormone (TH) homeostasis in humans and animals. However, the exact mechanism of thyroid dysfunction caused by PCBs is still unknown. In order to clarify the hypotheses that NADPH oxidase (NOX) and subsequent NF-*κ*B pathway may play roles in thyroid dysfunction, sixty Sprague-Dawley rats were randomly divided into four groups: control group, PCB153 treated (PCB) group, received apocynin with PCB153 treatment (APO + PCB) group, and drug control (APO) group. Serum thyroid hormone levels were evaluated. The morphological change of thyroid tissue was analyzed under the light and transmission electron microscopy. NOX2, 8-OHdG, and NF-*κ*B expression in the thyroid tissue was evaluated by immune-histochemical staining. Oxidative stress and inflammatory cytokines were detected. The following results were reduced after apocynin treatment: (1) serum thyroid hormone, (2) thyroid pathological injuries, (3) thyroid MDA, (4) thyroid ultrastructural change, (5) serum inflammatory cytokines, and (6) thyroid expression of NOX2, 8-OHdG, and NF-*κ*B. These results suggested that NOX inhibition attenuates thyroid dysfunction induced by PCB in rats, presumably because of its role in preventing ROS generation and inhibiting the activation of NF-*κ*B pathway. Our findings may provide new therapeutic targets for PCBs induced thyroid dysfunction.

## 1. Introduction

Polychlorinated biphenyls (PCBs) are a type of typical and widespread environmental endocrine disruptors which has been used in many industries. A large number of PCBs still exist in the environment due to their chemical stability and lipophilic properties. They affect human health by damaging multiple organs because of easily accumulating in adipose tissue and biomagnification in the food chain. PCBs have received wide attention due to their multiple range adverse effects on human health including hepatotoxicity, reproductive toxicity, developmental toxicity, immune suppression, carcinogenicity, and endocrine disruption such as the disruption of thyroid hormone (TH) homeostasis [1–4].

Thyroid hormones (THs) are essential for normal growth, development, and metabolism of organisms and an imbalance in TH levels could lead to a wide range of clinical conditions, such as fatigue, hair loss, poor memory, temperature intolerance, osteoporosis, and goiter [5]. Accumulating evidences have documented that thyroid function appears to be vulnerable to disruption by PCBs [6]. It is obvious that PCBs could interfere with thyroid hormone synthesis, secretion, transportation, and metabolism. Some researchers suggest that PCB153 could eliminate the biosynthesis of THs by influencing synthesis-associated proteins, such as the sodium iodide symporter (NIS), thyroid peroxidase (TPO), and thyroglobulin (Tg) [4].

Despite the fact that many possible mechanisms for PCBs-mediated disruption of TH homeostasis have been elucidated, one potential and important mechanism, NADPH oxidase (NOX) pathways, has been neglected. NOX produces a large amount of reactive oxygen species (ROS) and plays crucial roles in a variety of biological processes, such as host defense, signal transduction, hormone synthesis, mitogenic growth, apoptosis, angiogenesis, and oxidative modification of the extracellular matrix and extracellular proteins [7]. To date, four distinct groups of NOXs have been characterized including membrane-bound subunits gp22phox and gp91phox (also termed NOX2) as well as the cytosolic subunits including gp67phox and gp47phox [8].

In mammalian systems, PCBs and their metabolites have been shown to induce oxidative stress [4, 9]. Oxidative stress results from a disturbance of the normal cell balance between production of ROS and the capacity to neutralize their actions. Besides, NOX may produce a large amount of ROS mainly in pathogenesis of professional endocrinal cells such as pancreatic beta-cells [10]. Recent studies showed that NOX induced NF-*κ*B activation and inflammatory cytokine production in many chronic and acute inflammatory responses such as atherosclerosis and pancreatitis [8, 11]. Experimental studies demonstrated that NOX may participate in the pathophysiology of thyroid dysfunction mainly by regulating thyroid oxidative stress [12], so that treatments designed to modulate the production of ROS by NOX enzymes could provide new therapeutic approaches for some of these conditions.

We hypothesized that NOX may play significant roles in pathogenesis of thyroid dysfunction caused by PCBs. In this study, we aimed to evaluate (i) the involvement of NOX in thyroid dysfunction induced by PCBs and (ii) the protective effects of apocynin, a selective NOX inhibitor, on PCBs-induced thyroid injury of rats. In addition, we explored the underlying molecular mechanisms involved in these processes such as antioxidant and anti-inflammatory effects.

## 2. Experimental Section

### 2.1. Animals and Reagents

Male Sprague-Dawley rats, weighing 200 to 250 g, were obtained from the Center of Experimental Animals of Hubei Academy of Medical Sciences, Wuhan, China. The animals were kept at room temperature and 12 h light-dark cycles, and with free access to water. Rats in this study were maintained in accordance with the principles of the 1983 Declaration of Helsinki by the Ethics Committee of Wuhan University. Apocynin was obtained from Selleck Company (Houston, USA). PCB153 was obtained from DR Company (Augsburg, Germany) and corn oil was purchased from Sigma Aldrich Company (St. Louis, MO, USA). Antibodies were purchased from Abcam Company (Cambridge, UK).

### 2.2. Experimental Design

Sixty male rats were randomly assigned to four groups (*n* = 15 in each group): control group (C), PCB153 treated (PCB) group, received apocynin with PCB153 treatment (APO + PCB) group, and received apocynin only (APO) group. In the PCB group, rats received PCB153 dissolved in the corn oil injection for 5 consecutive days (32 mg/kg) intraperitoneally. In APO + PCB group, rats received apocynin (50 mg/kg) via femoral vein half an hour prior to PCB injection. In the control group, rats only received the vehicle (corn oil (4 mL/kg) and DMSO). In the APO group, rats received 50 mg/kg apocynin dissolved in the vehicle (10% DMSO v/v) and corn oil.

All rats were sacrificed 24 h after last injection. Blood was collected via heart puncture. Blood samples were centrifuging, and serum was stored at −20°C. The thyroid tissue was harvested and fixed in 4% PBS-buffered formaldehyde for histopathology and immunohistochemistry. The remaining part of thyroid tissues was immediately snap-frozen in liquid nitrogen and stored at −80°C for assay.

### 2.3. Biochemical Analysis

Serum concentration of the thyroid hormones (T3, T4, FT3, and FT4) was determined by radioimmunoassays (Beijing North Institute of Biological Technology, China). Serum levels of TNF-*α*, IL-1*β*, and IL-6 were quantified using specific ELISA kits according to the manufacturer's instructions (Biosource International, Nivelles, Belgium). Thyroid MDA and superoxide dismutase (SOD) level, a marker of lipid peroxidation, were measured using a commercial MDA, SOD assay kit (Nanjing Jiancheng Bioengineering Institute, Nanjing, China).

### 2.4. Histopathological Evaluation

Tissue samples were fixed in 4% paraformaldehyde in 0.1 M phosphate-buffered saline (PBS), embedded in paraffin, cut into 5 mm sections, and stained with hematoxylin and eosin (H&E). All tissue sections were examined microscopically for histopathological changes by an experienced histologist blinded to the study protocol. Thyroid gland damage was graded from 1 to 5 according to the following criteria: follicular size, colloid density, height of the follicular epithelium, mesenchymal fibrosis, and interstitial vascular proliferation [13]. The pathological scores were calculated by adding all scores from parameters described above.

### 2.5. Immunohistochemistry

After being deparaffinized and pretreated in citrate buffer, sections were incubated with normal goat serum for 15 min at room temperature. Sections were treated with rabbit anti-polyclonal antibody (NOX2, 8-OHdG, and NF-*κ*B) (Abcam) and negative control sections with normal rabbit serum and blank control sections with PBS overnight at 4°C. The secondary antibody, biotinylated anti-rabbit immunoglobulin, was applied for 15 min at room temperature and sections were rinsed in PBS. Peroxidase conjugated streptavidin was applied for 15 min, followed by diaminobenzidine (DAB) substrate for 10 min and hematoxylin for 5 min. Finally, sections were rinsed with water, dehydrated, cleared, and mounted with permanent mounting medium. Immunohistochemical micrographs were taken with the FSX-100 microscope camera system.

IHC staining was analyzed using Image Pro-Plus (version 6.0; Media Cybernetics, Silver Spring). Briefly, the positive staining area was selected as the area of interest (AOI). The area sum and integrated optical density (IOD) of the AOI were selected as the measurement parameters. The target protein expression was analyzed by comparing the IOD (NOX2 and 8-OHdG) and ratio of positive nuclear expression (NF-*κ*B) in different groups. Finally, statistical analysis of the mean expression index for each duplicate was performed.

### 2.6. Transmission Electron Microscopy (TEM)

Fresh thyroid specimens were fixed in a mixture of 2% formaldehyde and 2% glutaraldehyde in 0.1 mol/L cacodylate buffer (pH 7.4) overnight, postfixed in 2% osmium tetroxide in the same buffer. Ultrathin sections were cut on Leica EMUC7 ultramicrotome and stained with lead citrate, and changes of the thyroid follicle epithelial cells were examined in a HT7700 transmission electron microscope.

### 2.7. Statistical Analysis

Statistical analyses were carried out using SPSS statistical software (PASW Statistics for Windows, version 18.0). Data are expressed as mean ± SD. One-way analysis of variance (ANOVA) was used to investigate differences among the experimental groups. *P* < 0.05 is considered statistically significant.

## 3. Results

### 3.1. Effects of NOX Inhibition on PCB153-Induced Thyroid Dysfunction

**Figure 1:**
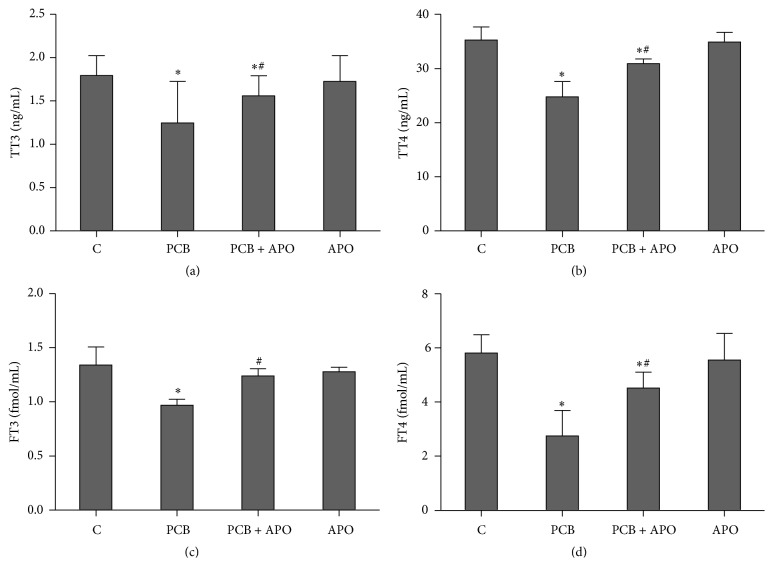
Effect of NOX inhibition on serum thyroid hormones: serum level of (a) T3; (b) T4; (c) FT3; (d) FT4. ^*∗*^ *P* < 0.05 versus control group. ^#^ *P* < 0.05 versus PCB group.

After the treatment with PCB153, serum levels of T3, T4, FT3, and FT4 were significantly decreased compared to control group (*P* < 0.05), indicating that rats were experiencing aggravated thyroid dysfunction. Apocynin induced significant increase on the level of serum thyroid hormones after PCB153 treatment (*P* < 0.05) (Figure 1).

### 3.2. Histological Effects of NOX Inhibition in Thyroid Tissues after PCB153 Treatment

**Figure 2:**
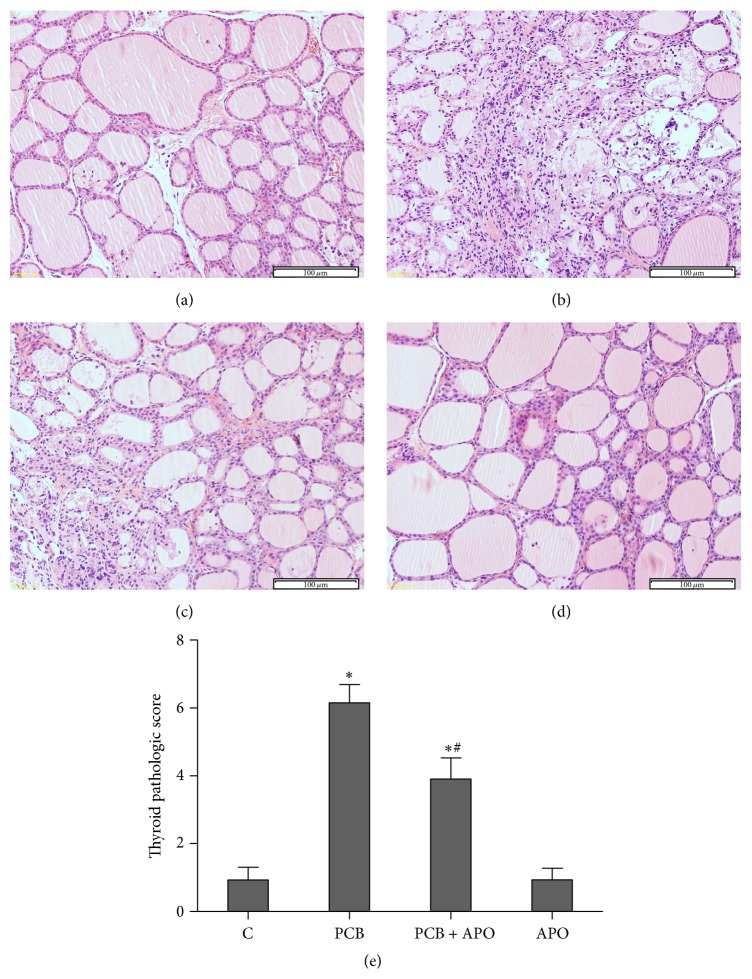
NOX inhibition attenuated thyroid morphologic changes of rats. (a) A representative figure from control group; (b) a representative figure from PCB group; (c) a representative figure from PCB + APO group; (d) a representative figure from APO group; (e) histological scores of thyroid tissue. ^*∗*^ *P* < 0.05 versus control group. ^#^ *P* < 0.05 versus PCB group (original magnification ×200).

Normal histological structure of thyroid tissue was observed in control (C) group and drug control (APO) group. In contrast, characteristic signs of thyroid injuries including hyperplasia and expansion of the follicles, shedding of epithelial cells, deficient luminal colloid, collapsed follicles, mesenchymal fibrosis, interstitial vascular proliferation, fibrinoid necrosis, or even disappearance of the follicular structure were observed in thyroid sections in PCB group (Figure 2(b)). However, the thyroid pathological changes improved and thyroid pathological grade reduced to a much lower level after pretreatment with apocynin in the PCB + APO group (Figures 2(c) and 2(e)).

### 3.3. Effects of NOX Inhibition on MDA Level and SOD Activity

**Figure 3:**
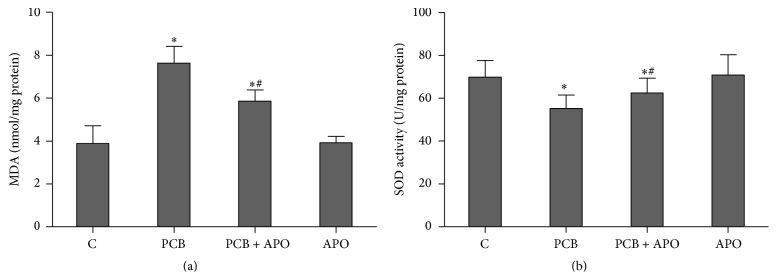
Effects of NOX inhibition on oxidative stress; (a) content of MDA; (b) SOD activity; ^*∗*^ *P* < 0.05 versus control group. ^#^ *P* < 0.05 versus PCB group.

To evaluate the oxidative stress induced by PCB153, MDA level and SOD activity in thyroid tissue were determined. Thyroid tissue MDA level in PCB group was elevated significantly compared to control and APO group (*P* < 0.05) (Figure 3). The elevation appeared to be significantly inhibited by apocynin pretreatment (*P* < 0.05). Moreover, similar changes were observed for SOD activity in thyroid tissues. This was found to be significantly depleted in PCB treated rats, probably as a result of oxidative stress processes. In contrast, treatment with apocynin has improved the activity of SOD in thyroid tissue (*P* < 0.05) (Figure 3).

### 3.4. Effects of NOX Inhibition on Inflammatory Cytokines

**Figure 4:**
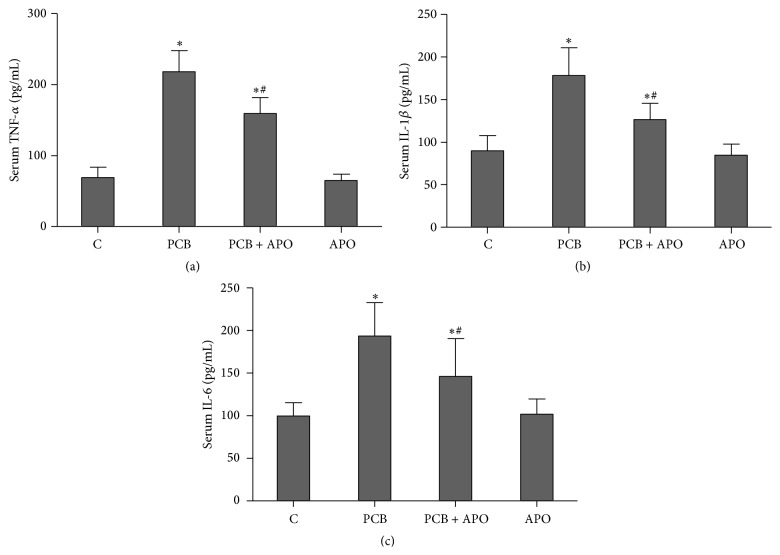
Effects of NOX inhibition on proinflammatory cytokines production; (a) TNF-*α*; (b) IL-1*β*; (c) IL-6; ^*∗*^ *P* < 0.05 versus control group. ^#^ *P* < 0.05 versus PCB group.

Serum concentrations of proinflammatory cytokines were analyzed to obverse anti-inflammatory effect of apocynin after PCB153 treatment. As illustrated in Figure 4, serum levels of TNF-*α*, IL-1*β*, and IL-6 after PCB153 treatment were increased compared to control group (*P* < 0.05). However, there were significant decreases in PCB + APO group compared to PCB group (*P* < 0.05).

### 3.5. Effects of NOX Inhibition on Expression of 8-OHdG and NF-*κ*B in Thyroid Tissue

#### 3.5.1. Effect of Apocynin on NOX2 Expression in the Thyroid Tissue

**Figure 5:**
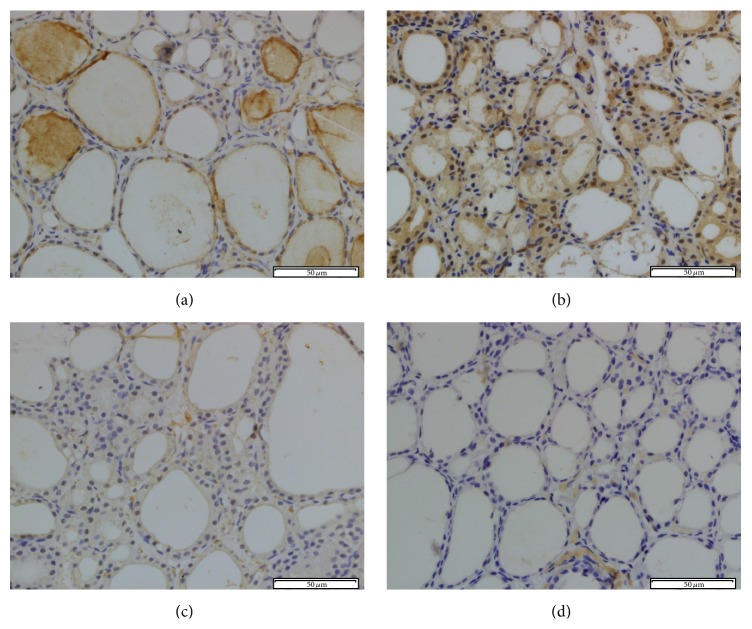
NOX2 immunohistochemical staining in rat thyroid tissue. The immunohistochemical localization of NOX2 appears as brown staining. (a) Control group; (b) PCB group; (c) PCB + APO group; (d) APO group (original magnification ×400).

Immunohistochemical staining of NOX2 was used to evaluate whether PCB153 can induce NOX2 expression and apocynin successfully blocked PCB153-induced NOX2 expression in the thyroid tissue. Thyroid tissue sections from the control and APO group rats showed little expression of NOX2 (Figures 5(a) and 5(d)). NOX2 immunoreactivity was markedly enhanced in PCB rats (Figure 5(b)) and expression of NOX2 was reduced (Figure 5(c)) in the thyroid tissue that had undergone apocynin pretreatment. Quantitative analysis revealed that IOD value of NOX2 increased significantly in PCB group (*P* < 0.05). IOD value of NOX2 decreased after apocynin treatment (*P* < 0.05) (Figure 8(a)).

#### 3.5.2. Effect of Apocynin on 8-OHdG Expression in the Thyroid Tissue

**Figure 6:**
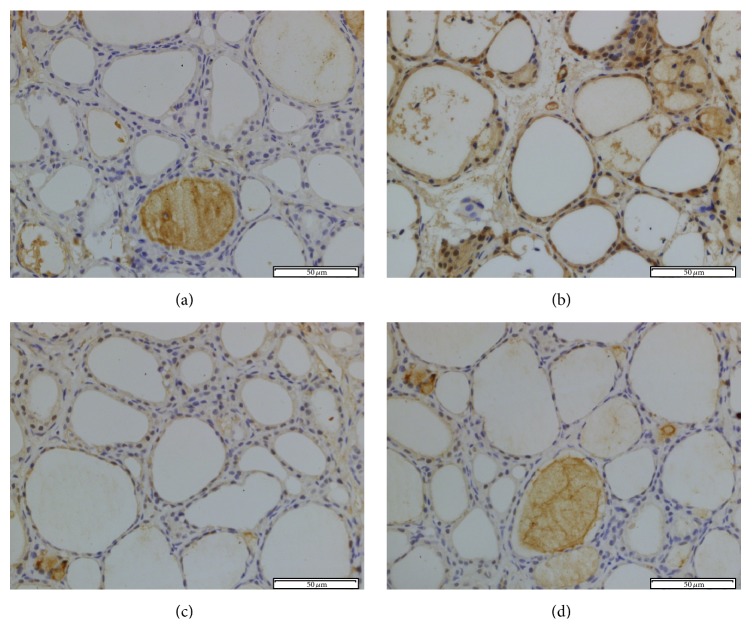
8-OHdG immunohistochemical staining in rat thyroid tissue. The immunohistochemical localization of NOX2 appears as brown staining. (a) Control group; (b) PCB group; (c) PCB + APO group; (d) APO group (original magnification ×400).

To investigate the effect of NOX inhibition on PCB153-induced oxidative stress, immunohistochemical staining of 8-OHdG was performed in all groups. 8-OHdG is a ROS-induced DNA damage marker and 8-OHdG-positive signals were much denser and mainly detected in the nuclei of PCB153 treated follicular epithelial cells (Figure 6(b)). The signals were decreased by apocynin treatment significantly (Figure 6(c)). 8-OHdG immunoreactivity was very low in thyroid tissue from both control and APO group (Figures 6(a) and 6(d)). Quantitative analysis revealed that IOD value of 8-OHdG increased significantly in PCB group (*P* < 0.05). IOD value of 8-OHdG decreased after apocynin treatment (*P* < 0.05) (Figure 8(b)).

#### 3.5.3. Effect of Apocynin on NF-*κ*B Expression in the Thyroid Tissue

**Figure 7:**
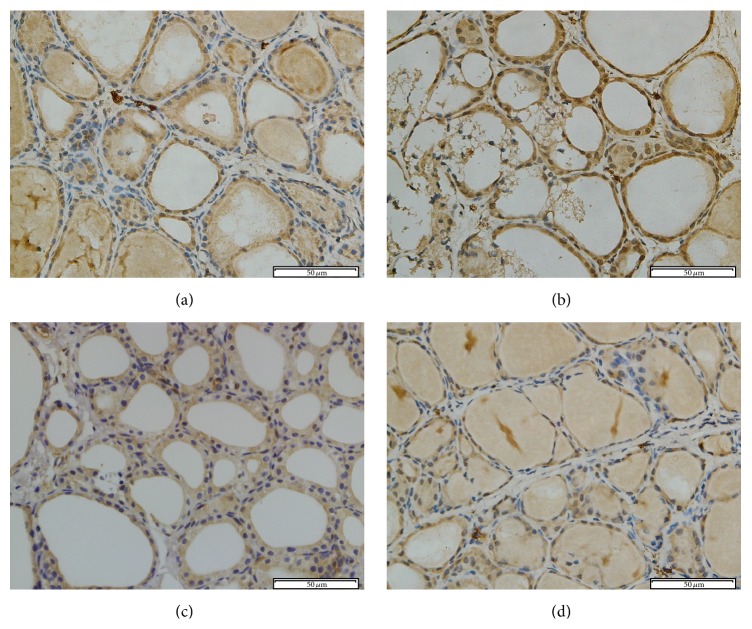
NF-*κ*B immunohistochemical staining in rat thyroid tissue. The immunohistochemical localization of NF-*κ*B appears as brown staining. (a) Control group; (b) PCB group; (c) PCB + APO group; (d) APO group (original magnification ×400).

**Figure 8:**
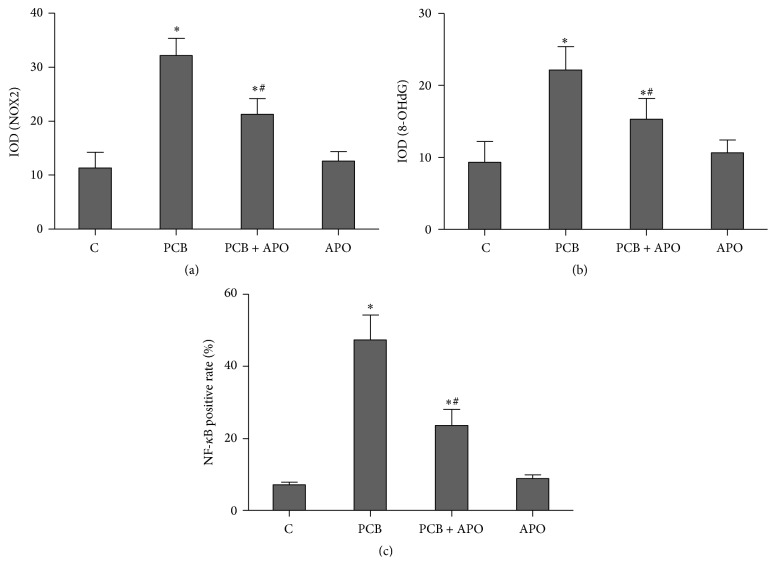
Immunohistochemical analysis. (a) NOX2; (b) 8-OHdG; (c) NF-*κ*B; ^*∗*^ *P* < 0.05 versus control group. ^#^ *P* < 0.05 versus PCB group.

The expressions of NF-*κ*B were concentrated mainly in the cytoplasm in the control and APO group (Figures 7(a) and 7(d)). In PCB treated group, NF-*κ*B immunoreactivity was highly expressed in nucleus (Figure 7(b)). However, a marked decrease in NF-*κ*B staining was found in the nucleus with the apocynin pretreatment group (Figure 7(c)). Quantitative analysis revealed that ratio of NF-*κ*B positive nucleus in thyroid section from PCB group was increased significantly compared to control group (*P* < 0.05). However, ratio of NF-*κ*B positive nucleus decreased after apocynin treatment (*P* < 0.05) (Figure 8(c)).

### 3.6. Ultrastructural Changes of Thyroid Follicular Cells under Transmission Electron Microscopy (TEM)

**Figure 9:**
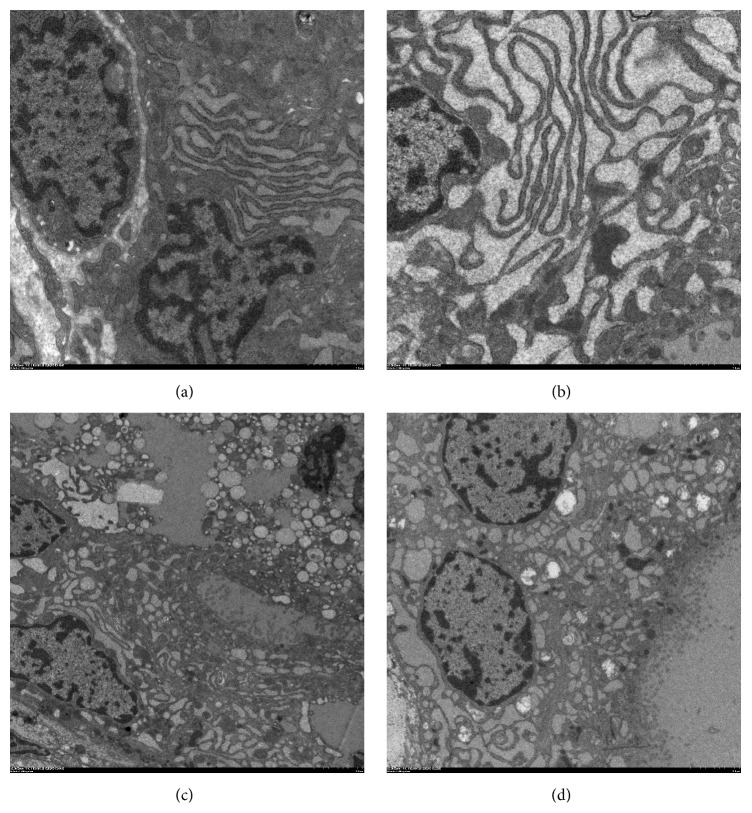
TEM comparison of thyroid follicular cells architecture. (a) A TEM analysis from the control group showing that the follicular cells contain well-developed parallel cisternae of ER, mitochondria, and large moderate electron dense cytoplasmic colloid droplets. Apical lateral surfaces of follicular cells show tight junctions (magnification, ×5,000); (b) TEM analysis from the PCB group showed that the follicular cells contained many vacuoles and few microvilli. Follicular cells have euchromatic nuclei with peripheral rim of heterochromatin and markedly dilated cisternae of ER (magnification, ×5,000); (c) in PCB group, corrugated heterochromatic follicular cells nuclei and desquamated follicular cells within follicular lumen are noticed. The general loss of subcellular organization and cellular contents is also found (magnification, ×2,000); (d) TEM analysis from the PCB + APO group showing that the follicular cells cytoplasm contains moderately dilated cisternae of ER, Golgi saccules, and apical electron dense granules (magnification, ×2,000).

TEM analysis of thyroid tissue demonstrated that follicular epithelial cells in control group showed normal morphology of nucleus, mitochondria, endoplasmic reticulum (ER), ribosomes, and other cellular organelles (Figure 9(a)). In PCB treated rats, most of the follicular cells had corrugated heterochromatic nuclei and some of them were surrounded by fragmented dilated ER. Swollen mitochondria and markedly dilated ER cisternae were present in the basal pole (Figure 9(b)). There was a general loss of subcellular organization and of cellular contents (Figure 9(c)). TEM examination of the section from PCB + APO group has revealed that many follicular cells had euchromatic nuclei with peripheral rim of heterochromatin. Their cytoplasm contained moderately dilated cisternae of ER, mitochondria, Golgi saccules, and apical electron dense granules. These suggested that ultrastructural damage of follicular cells was ameliorated by apocynin treatment (Figure 9(d)).

## 4. Discussion

The results of the current study demonstrate that exposure to PCB153 could severely damage thyroidal structure, dramatically decreasing the concentration of serum thyroid hormones. In addition, it also induces release of inflammatory cytokines and oxidative stress. Besides, NOX expression in thyroid follicles was markedly increased which means NOX would be one significant mechanism of PCB153-induced disruption of TH homeostasis. Pretreatment with the NOX inhibitor apocynin attenuates (1) serum TH level; (2) morphological change of thyroid follicles; (3) ultrastructural change of thyroid follicular cells; (4) proinflammatory cytokine production; (5) MDA; (6) expression of NOX2, NF-*κ*B, and 8-OHdG. All of these observations suggest that NOX may participate in the process of thyroid dysfunction induced by PCB153 and treatment with NOX inhibition by apocynin exerts potent anti-inflammatory and antioxidative stress effects and ameliorates the degree of PCB153-induced thyroid injury in rats.

A growing number of reports have demonstrated that PCBs and their metabolites have been shown to induce oxidative stress [4, 9]. Under physiological condition, tissues contain various endogenous antioxidant enzymes like GSH and SOD, which scavenge reactive oxygen species (ROS) and prevent lipid peroxidation. When ROS generation exceeds the antioxidant capacity of cells, oxidative stress develops. GSH-PX and SOD as the ROS scavengers are depleted; MDA as a product of lipid peroxidation and 8-OHdG as a ROS-induced DNA damage marker are accumulated. The overproduced ROS was able to directly or indirectly damage the thyroid tissues and result in thyroid dysfunction and histological changes. Our results showed that the activity of SOD was significantly increased when using the apocynin treatment in PCB administered rat. Oppositely, the MDA level and expression of 8-OHdG were decreased. These results combined with the morphologic change of thyroid tissue indicate that apocynin reduces the level of ROS by inhibiting the NOX further and ameliorating thyroid oxidative damage.

Evidence suggests that inflammatory cytokines infiltration such as TNF-*α*, IL-1*β*, and IL-6 is not uncommon in PCB induced cardiovascular inflammation [14–16]. Possible mechanism of that phenomenon is that ROS production induced by PCB caused deregulation of cellular redox status. It can lead to upregulation of nuclear factor kappa B (NF-*κ*B) and subsequently the induction of multiple proinflammatory gene products including chemokines, cytokines, and cellular adhesion molecules [17]. However, major contributions of NOX during inflammatory reaction play a role as an inflammatory stimulator activating the leukocyte system which then can produce and release a variety of secondary inflammatory mediators [18]. Other studies suggested that NOX involves the pathogenesis of various inflammatory diseases such as diabetes associated vascular inflammation [19], adipose tissue inflammation and insulin resistance [20], and acute and chronic inflammation of the colon [21]. Data from the present study demonstrated increase of these cytokines after PCB administration. However, their serum levels were reduced in rats treated with apocynin. These results were consistent with previous studies that apocynin attenuates experimental airway inflammation via inhibition of inflammatory cytokines production [22].

Some studies indicated that PCB may induce NF-*κ*B activation and induce subsequent inflammatory process [23]. It is obvious that ROS produced by NOX can also induce expression of NF-*κ*B and MAPK pathway in addition to the cytokines production. Then, we hypothesize that by NOX mediated NF-*κ*B activation may involve PCB-induced disruption of thyroid hemostasis. NF-*κ*B is a transcription factor necessary for the transcription of many proinflammatory mediators such as cytokines, chemokines, and oxygen derived free radicals. In quiescent cells, NF-*κ*B is present in the cytosol complexed with I-*κ*B. The phosphorylation of I-*κ*B on serines within the amino-terminal domain results in the dissociation and translocation of NF-*κ*B to the nucleus and initiates gene transcription [24]. Recent studies demonstrated that NOX dependent NF-*κ*B activation may play a major role in inflammation in rat vascular smooth muscle and inhibition of NOX leads to reduction of inflammation via NF-*κ*B dysregulation [25]. Another study suggested that NOX may induce NF-*κ*B activation in acute lung inflammation [26]. In our present study, we found that the translocation of activated NF-*κ*B into the nucleus was significantly increased in thyroid tissue of PCB153 exposed rat. However, after the treatment of apocynin, the nuclear expression of NF-*κ*B was decreased. These results suggested that the activation of NF-*κ*B could be inhibited by apocynin via blocking the NOX. This finding was supported by other studies that apocynin attenuated isoproterenol induced brain inflammation by inhibiting NF-*κ*B activation and ER stress [27].

In conclusion, the present study showed that pretreatment with apocynin, a NOX inhibitor, reduced the severity of PCB153-induced thyroid injury by reducing inflammation and oxidative stress and downregulated NF-*κ*B expression. Thus, apocynin treatment could ameliorate PCB-induced thyroid injury and this would be one novel and significant therapeutic target for PCBs-mediated thyroid disfunction.
